# Natural language processing to identify suicidal ideation and anhedonia in major depressive disorder

**DOI:** 10.1186/s12911-025-02851-w

**Published:** 2025-01-13

**Authors:** L. Alexander Vance, Leslie Way, Deepali Kulkarni, Emily O. C. Palmer, Abhijit Ghosh, Melissa Unruh, Kelly M. Y. Chan, Amey Girdhari, Joydeep Sarkar

**Affiliations:** 1 Holmusk Technologies, Inc, 54 Thompson St, New York, NY 10012 USA; 2 KKT Technologies, Pte. Ltd, Blk 71, Ayer Rajah Crescent, #06-07/08/09 and #07-08/09, Singapore, 139951 Singapore; 3 Holmusk Europe, Ltd, 414 Linen Hall, 162-168 Regent St, London, W1B 5TE UK

**Keywords:** Natural language processing, Electronic health records, Suicidal ideation, Anhedonia, Major depressive disorder

## Abstract

**Background:**

Anhedonia and suicidal ideation are symptoms of major depressive disorder (MDD) that are not regularly captured in structured scales but may be captured in unstructured clinical notes. Natural language processing (NLP) techniques may be used to extract longitudinal data on suicidal behaviors and anhedonia within unstructured clinical notes. This study assessed the accuracy of using NLP techniques on electronic health records (EHRs) to identify these symptoms among patients with MDD.

**Methods:**

EHR-derived, de-identified data were used from the NeuroBlu Database (version 23R1), a longitudinal behavioral health real-world database. Mental health clinicians annotated instances of anhedonia and suicidal symptoms in clinical notes creating a ground truth. Interrater reliability (IRR) was calculated using Krippendorff’s alpha. A novel transformer architecture-based NLP model was trained on clinical notes to recognize linguistic patterns and contextual cues. Each sentence was categorized into one of four labels: (1) anhedonia; (2) suicidal ideation without intent or plan; (3) suicidal ideation with intent or plan; (4) absence of suicidal ideation or anhedonia. The model was assessed using positive predictive values (PPV), negative predictive values, sensitivity, specificity, F1-score, and AUROC.

**Results:**

The model was trained, tested, and validated on 2,198, 1,247, and 1,016 distinct clinical notes, respectively. IRR was 0.80. For anhedonia, suicidal ideation with intent or plan, and suicidal ideation without intent or plan the model achieved a PPV of 0.98, 0.93, and 0.87, an F1-score of 0.98, 0.91, and 0.89 during training and a PPV of 0.99, 0.95, and 0.87 and F1-score of 0.99, 0.95, and 0.89 during validation.

**Conclusions:**

NLP techniques can leverage contextual information in EHRs to identify anhedonia and suicidal symptoms in patients with MDD. Integrating structured and unstructured data offers a comprehensive view of MDD’s trajectory, helping healthcare providers deliver timely, effective interventions. Addressing current limitations will further enhance NLP models, enabling more accurate extraction of critical clinical features and supporting personalized, proactive mental health care.

**Supplementary Information:**

The online version contains supplementary material available at 10.1186/s12911-025-02851-w.

## Background

Suicide is a major public health concern, with more than a million people reported to die by suicide every year [1], and many more who have engaged in non-fatal suicidal behaviors, suicidal ideation (SI), or attempts. Suicidal thoughts and behaviors are common among patients with major depressive disorder (MDD), with lifetime prevalence rates of 37.7% for SI, 15.1% for suicidal planning, and 23.7% for suicidal attempts [2]. Research on suicide has been limited by factors including reporting biases and the relative rarity of the event, however, the use of large health information databases, such as electronic health records (EHR), has been increasingly recognized as valuable in progressing this crucial area of research [3–5].

Anhedonia represents another critical facet of MDD that warrants further research. Anhedonia, characterized by the pervasive inability to experience pleasure or interest in previously enjoyable activities, serves as one of the two primary diagnostic criteria for MDD [6]. Anhedonia has been studied as an important symptom or behavioral domain of depressive disorders and is a pathological biomarker for predicting treatment outcomes and assisting treatment plan development [7, 8]. Individuals with MDD also face an elevated risk of various physical illnesses [9]. This high comorbidity is linked to poorer health outcomes, lower treatment adherence, increased mortality, and higher healthcare utilization and costs [9]. Previous research has deployed natural language processing (NLP) to extract pertinent data from unstructured text fields to identify patients with MDD [10], and extract information such as diagnosis, medication, symptoms, and adverse events [11]. The application of NLP models to EHR data could enrich data sources to phenotype patients, gain a more comprehensive understanding of clinical practice, guide treatment selection, and identify areas of unmet need.

The application of NLP to phenotype patients is of particular interest as there is emerging evidence of the crucial role of distinguishing between patients with MDD who do and do not have anhedonia [12]. This distinction may inform targeted treatments, given the involvement or dysfunction of distinct brain circuits (e.g., striatal hypoactivation) in these phenotypes [13, 14]. Furthermore, compelling evidence at the structural and functional levels suggests that phenotypic segmentation leads to the identification of markedly different archetypes among patients [12, 15, 16]. Leveraging NLP techniques to extract insights from unstructured clinical notes may facilitate further phenotypic segmentation of patients in real-world settings.

Earlier studies leveraging EHR data have primarily utilized structured data (e.g., demographic information, diagnostic codes, and cause-of-injury codes) to ascertain suicide rates and risk factors [17, 18]. However, the practices around documenting suicidal thoughts and behaviors vary widely across service providers and clinicians, with previous research finding only 3% of patients with ideation and 19% of patients with attempt having a corresponding structured codes [19].

Large volumes of routinely collected unstructured EHR data have been used to track longitudinal patient trajectories in oncology [20] and metabolic diseases [21, 22]. Similarly, the application of NLP techniques in mental health and psychiatry could provide unique insights in a field traditionally plagued with limited administration of standardized measurements and where data on symptomology and treatment progress are almost exclusively found in clinicians’ unstructured notes. The use of NLP text-mining techniques on psychiatric data thus could allow for the extraction of longitudinal data on critical types of clinical information, including suicidal behaviors and anhedonia. Though the application of NLP in psychiatry is still considered to be in its infancy, there has been a large increase in studies on this topic over the past two decades [23–28]. However, a recent review of 110 relevant NLP text-mining studies suggested that the majority of datasets used to train the machine learning models only contained hundreds or thousands of documents despite the availability of much larger datasets due to the annotation bottleneck faced by supervised machine learning algorithms [29].

Consequently, this study was conducted to create a clinical consensus via an iterative process to clearly identify when a medical note refers to the presence of anhedonia or suicidal thoughts and behaviors. The study aimed to use these clinically annotated notes to build upon extant NLP text-mining techniques within psychiatry by presenting a fit-for-purpose approach to identifying anhedonia and SI from real-world data. The model developed and used in our study was initially pre-trained on more than 180,000 clinical documents detailed in Kulkarni et al., 2023 [30]. This additional layer of validation was included to overcome the challenges of unstandardized vocabulary in this therapeutic area through engaging a team of mental health clinicians to participate in the annotation process. Finally, this study also aimed to demonstrate the use of NLP text-mining techniques to enrich real-world data sources to extract meaningful insights into real-world clinical practice.

## Methods

### Study design

This study developed and evaluated an NLP model for identifying anhedonia and SI in patients with MDD. The study employed a retrospective analysis of clinical notes to develop and validate the NLP model.

### Data source

This study leveraged EHR-derived, de-identified data from the NeuroBlu Database (version 23R1), a longitudinal behavioral health real-world database comprising both structured and unstructured patient-level clinical data [22]. Data for the current study were sourced from a single data partner, a large community-based mental health network that comprises over 70 sites of care in a densely populated metropolitan area of the state of Texas. The inclusion criteria consisted of patients with a recorded diagnosis of MDD selected based on International Classification of Diseases (ICD)-9-Clinical Modification (CM) and ICD-10-CM diagnostic codes recorded in EHRs.

### Clinical annotation process

**Fig. 1:**
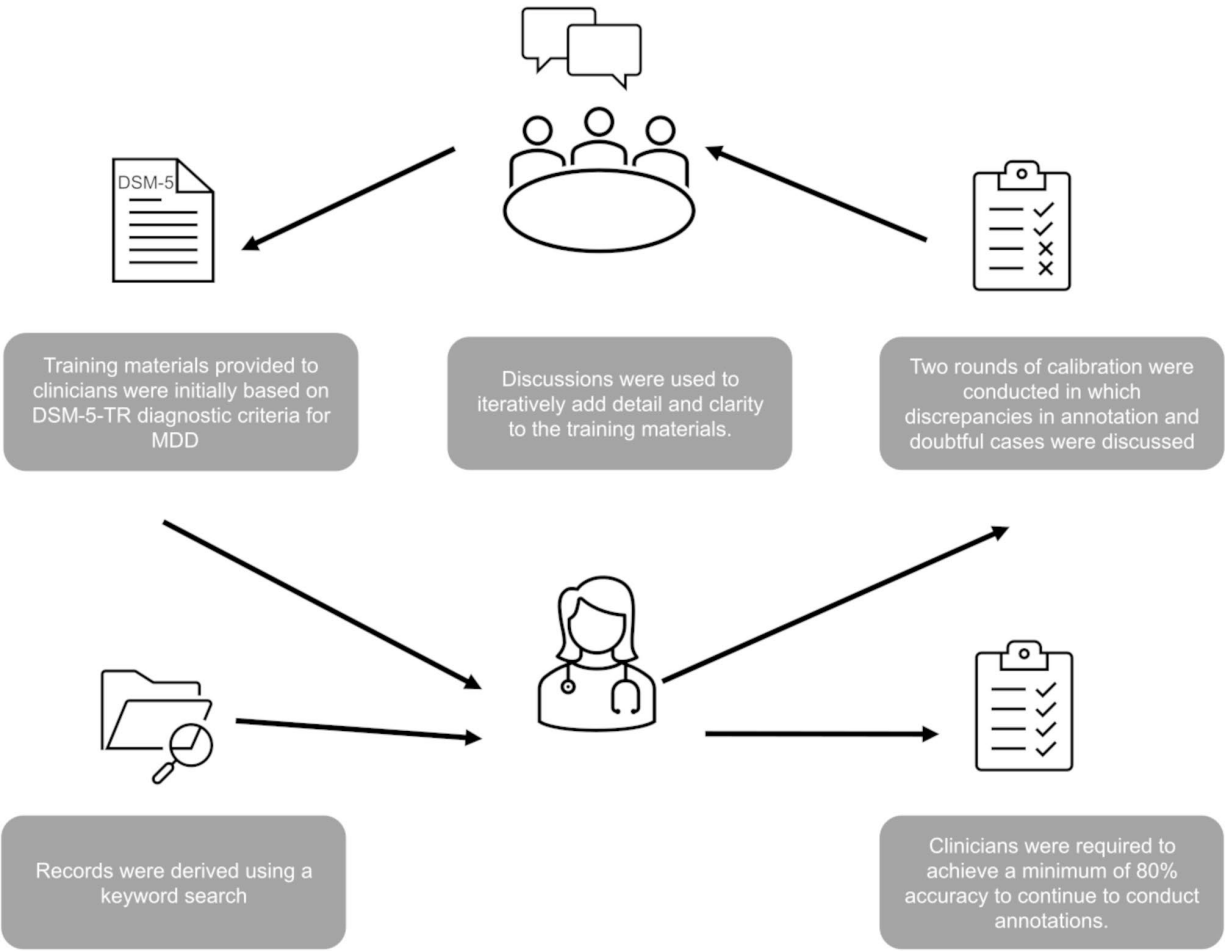
Clinical annotation process used to create a ground truth dataset. **Abbreviations:** DSM-5-TR, Diagnostic and Statistical Manual of Mental Disorders, Fifth Edition, Text Revision; MDD, major depressive disorder

An outline of the clinical annotation process for producing a ground truth dataset for finetuning and verifying the model is shown in Fig. 1. A sample of 5,000 MDD specific clinical free text extracts were annotated by a team of six United States-licensed mental health clinicians (e.g., psychiatrists, clinical psychologists, mental health counsellors, clinical social workers, etc.). The records were first derived using a keyword search to obtain a subset of text segments with a high number of target data present (see Additional File 1 for the keywords list).

The training materials provided to clinicians to guide annotations were initially based on Diagnostic and Statistical Manual of Mental Disorders, Fifth Edition, Text Revision clinical criterion regarding suicidality [6]. The training procedure for clinicians was an iterative process and included synchronous calls, personalized feedback, and a competency evaluation. Clinicians were required to achieve a minimum of 80% accuracy during the competency evaluation to continue annotations.

Clinicians were provided with training materials and sample of sentences extracted from a clinical note and were tasked to annotate whether the sentence contained the presence of either anhedonia, SI without or unknown intent or plan, or SI with intent or plan. Based on the clinician’s annotations, each sentence could be categorized into one of four labels: (1) anhedonia; (2) SI without intent or plan; 3 SI with intent or plan; or 4) absence of SI or anhedonia. Temporality was not considered by the model (i.e., the model did not consider whether symptoms were described as current or historic), thus each sentence could have labels 1, 2, and 3, but 4 was only given if no other labels were present. For example, a sentence could be labelled as both 2 and 3 if suicidal plan were mentioned in the past and the patient had active ideation during the current visit.

Two rounds of calibration were conducted and discrepancies in annotation and doubtful cases were discussed by eight clinicians. These discussions were used to iteratively add detail and clarity to the training materials. Krippendorff’s alpha was calculated for each round of calibration to demonstrate the improvement in interrater reliability (IRR). An alpha of above 0.8 is considered reliable [31]. Following the calibration rounds two clinical annotators were lost from the study, therefore there were six annotators used for the remaining annotations. The 5,000 clinically annotated patient records were checked for duplicates and note length; duplicates and notes shorter than 100 words were removed (see Additional File 1). The remaining 4,461 clinical notes were divided into training (*n* = 2,198), testing (*n* = 1,247), and validation (*n* = 1,016) subsets using stratified random sampling techniques. The models were trained on the training set and fine-tuned using the test set to optimize performance.

### Model development

The development of the NLP model involved the use of deep learning and transformer architecture, which were explained in a previous publication [30]. The model was pretrained on a sample of 189,676 unique clinician notes taken between 2016 and 2021 from 427,777 patients receiving mental health treatment. Following pre-training the model was fine-tuned via clinical annotation by a team of mental health clinicians.

### Assessment of utility of NLP model for extracting clinical data

The NeuroBlu Database contains both structured and unstructured patient-level clinical data. We assessed the potential utility of the model by calculating the availability of data pertaining to suicidal symptoms and anhedonia from structured data sources and compare these with a combination of structured data and data extracted from unstructured clinical notes using our model. This analysis was conducted on all patients with an MDD diagnosis from the data source that was originally used to develop the model. This was to gain an insight into how the application of the model may enrich the clinical utility of an EHR database. Further details are provided in Additional File 1.

Our model enhances data granularity by extracting nuanced symptom-specific insights, such as anhedonia and suicidal ideation, from unstructured clinical notes. These additional data points enrich structured data fields by capturing symptom nuances and progression often absent in standard records. Data enhancement was calculated as the percentage increase in analyzable data points within structured data fields after the inclusion of NLP-derived data. For instance, a 17% enhancement for anhedonia reflects new patients identified with symptom-specific information beyond existing structured fields from standardized questionnaires. This process ensures the model not only complements but also extends the utility of structured EHR data. To calculate the data enhancement of net new patients and visits with relevant extracted symptoms, we first calculated the raw counts added through the incorporation of the model on top of the existing structured data. We then adjusted these counts by multiplying by the respective F1 score of each model calculated during the validation step to account for potential erroneous cases generated by the model. This resulted in the total number of unique patients and total number of unique visits in which the presence or absence of the depressive symptoms could be successfully identified.

### Statistical analysis

The model’s performance during both training and validation was assessed using standard performance matrices such as positive predictive values (PPV), negative predictive values (NPV), sensitivity, specificity, F1-score, and area under the receiver operating characteristic (AUROC). An F1-score ranges from 0 to 1 and is considered good when the value is > 0.8 [32]. Interpreting these results collectively provides a comprehensive assessment of the model’s performance. High values of PPV, NPV, sensitivity, specificity, and F1-score suggest that the model is effective in making accurate predictions for both positive and negative cases. An AUROC value of 0.5 indicates no discrimination, and higher values signify better-than-random performance, with AUROC values of 0.7–0.8, 0.8–0.9, and > 0.9 indicating acceptable, excellent, and outstanding discriminatory performance, respectively [33].

### Ethical considerations

Institutional review board approval of the study protocol, including a waiver of Health Insurance Portability and Accountability Act authorization, was obtained prior to study conduct, and covers data originating from all sites represented.

## Results

### Study sample

A cohort of 3,305 patients were included who had a recorded MDD diagnosis and a documented clinical notes containing information related to anhedonia and suicidality, the patients’ demographic characteristics, and comorbidities are shown in Table 1. Each note encapsulates the clinical narrative for a single patient during a specific encounter.

**Table 1:** Demographic characteristics of patients with MDD

Demographic characteristic	Cohort (*N* = 3,305)
Mean (SD) age at time of first MDD diagnosis	33.3 (14.8)
Sex, *n* (%)	
Female	1703 (51.5)
Male	1600 (48.4)
Other	2 (0.1)
Race, *n* (%)	
White	1646 (49.8)
Black or African American	1256 (38.0)
Unknown	335 (10.7)
Other	22 (1.5)
Ethnicity, *n* (%)	
Not Hispanic or Latino	1558 (47.1)
Hispanic or Latino	802 (24.3)
Unknown	945 (28.6)
Psychiatric comorbidity at any time in EHR, *n* (%)	
Substance-related disorders	1734 (39.1)
Bipolar disorder	425 (9.6)
Schizoaffective disorders	349 (7.9)
Post-traumatic stress disorder	265 (6.0)
Adjustment disorders	236 (5.3)
Generalized anxiety disorder	218 (4.9)
Personality disorders	165 (3.7)
Attention-deficit hyperactivity disorders	162 (3.7)
Schizophrenia	137 (3.1)
Conduct disorders	131 (3.0)
Panic disorder	102 (2.3)
Mental disorders due to known physiological conditions	88 (2.0)
Intellectual disabilities	79 (1.8)
Behavioral syndromes associated with physiological disturbances and physical factors	79 (1.8)
Pervasive and specific developmental disorders	79 (1.8)
Phobic anxiety disorders	59 (1.3)
Obsessive-compulsive disorder	55 (1.2)
Dysthymic disorder	40 (0.9)
Impulse disorders	28 (0.6)

**Abbreviations:** EHR, electronic health record; MDD, major depressive disorder; SD, standard deviation

**Note:** All demographic characteristics were recorded at first entry to EHR, except age, which was recorded at the time of first diagnosis, and psychiatric comorbidities which were recorded at any time in the EHR

### Annotation calibration

In the initial calibration round a subset of 94 sentences were independently annotated by eight annotators. Krippendorff’s alpha for the first round of calibration was 0.47, indicating a low level of agreement between the annotators. To assess the impact of calibration and training, a different subset of 78 sentences was selected. This subset underwent the second round of calibration with the same eight annotators. The annotations were compared with those from the first round. Krippendorff’s alpha for round two calibration exhibited a notable increase, reaching 0.80, indicating improved agreement.

### Performance metrics

The model’s ability to accurately extract anhedonia and suicidal symptom data from clinical notes is shown in Table 2. The performance matrices for both the testing and validation cohorts indicate the NLP model achieved a high degree of accuracy in identifying depressive symptom data.

**Table 2:** Model accuracy for testing and validation datasets

**Symptom**	**Testing** **(Notes = 1,247)**					
	**Sensitivity**	**Specificity**	**PPV**	**NPV**	**F1**	**AUROC**
Anhedonia	0.98	0.90	0.98	0.99	0.98	0.99
SI with plan or intent	0.89	0.89	0.93	0.98	0.91	0.96
SI without plan or intent	0.92	0.86	0.87	0.99	0.89	0.93
**Symptom**	**Validation** **(Notes = 1**,**016)**					
	**Sensitivity**	**Specificity**	**PPV**	**NPV**	**F1**	**AUROC**
Anhedonia	0.99	0.81	0.99	0.99	0.99	0.99
SI with plan or intent	0.94	0.77	0.95	0.98	0.94	0.97
SI without plan or intent	0.92	0.83	0.87	0.98	0.88	0.93

Abbreviations: AUROC, area under the receiver operating characteristic; NPV, negative predictive value; PPV, positive predictive value; SI, suicidal ideation

### Clinical utility – data availability

**Fig. 2:**
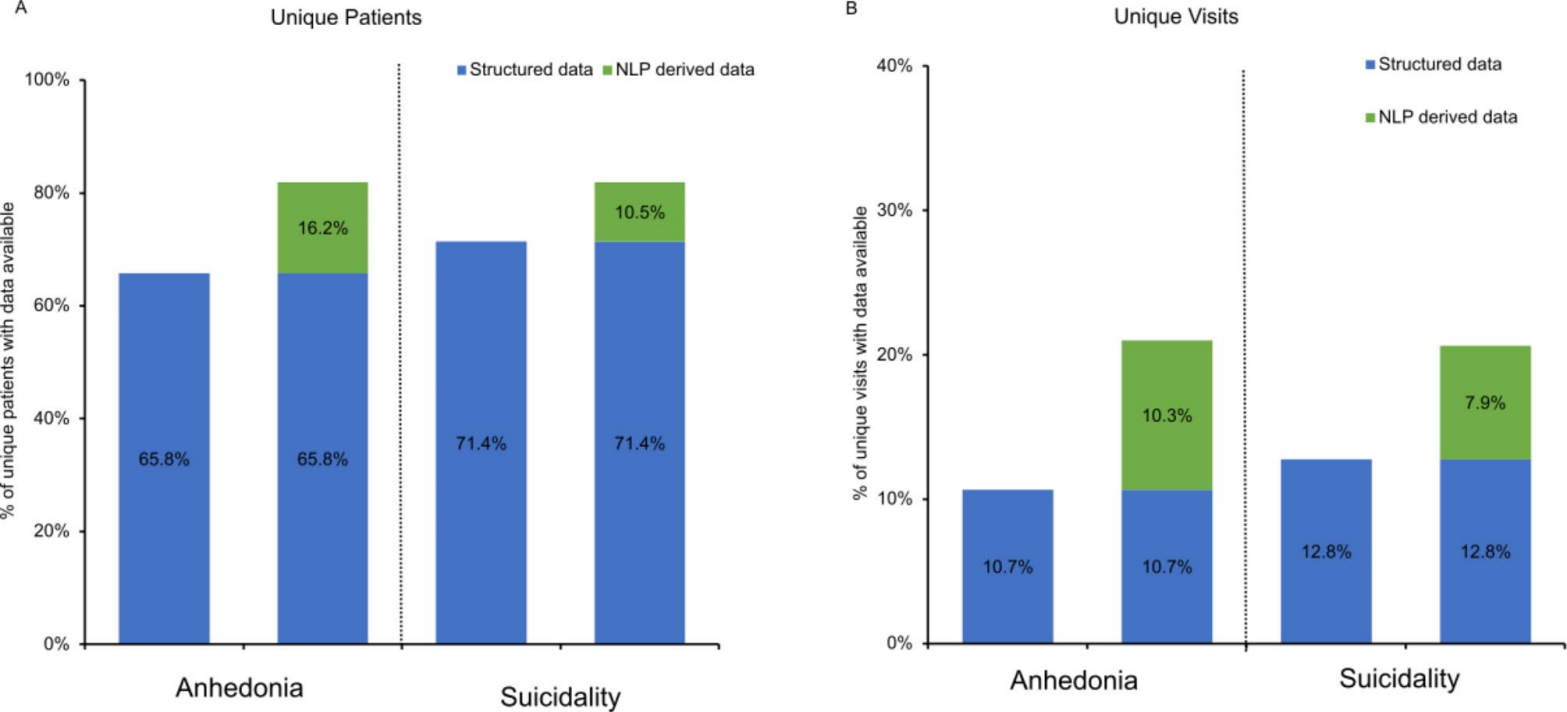
Change in availability of unique visits and patients before and after applying the NLP model. **Note:** NLP derived data was corrected for model accuracy (F1 Score). **Abbreviations:** NLP, natural language processing

The increase in data availability for both unique visits and unique patients is shown in Fig. 2. There was a substantial increase in data availability within the EHR on a patient level, and even more so on a visit level. The proportion of the total 39,034 unique patients with MDD who had data available for anhedonia and suicidality increased from 65.8% and 71.4 to 82.0% and 81.9%, respectively. The ability of the model to enrich the dataset was also limited as only 56.0% (n = 22,205) of patients had clinical notes available in the EHR record. Of the total 458,233 unique visits, 15.5% (n = 71,129) had notes available in the EHR. The visit data available for anhedonia and suicidality increased from 10.7% and 12.8 to 21.0% and 20.7%, respectively. This demonstrates the potential utility of our model to enable the extraction of additional clinical features, leading to a notable increase in availability of clinically relevant information related to MDD. This ability to extract nuanced details from free-text EHRs not only enriches the dataset but also enables earlier identification of symptom patterns and risk factors, facilitating a more proactive approach to care. By transforming unstructured data into actionable insights, the model supports clinicians in monitoring symptom progression and making timely interventions.

## Discussion

The current study describes the clinical validation process for extracting data on anhedonia and suicidal symptoms from unstructured clinical notes of patients with MDD. Our rigorous process ensured accurate and precise capture of relevant data among the many ways anhedonia and suicidal symptoms may be documented, as evidenced from the high-performance metric scores during the training and validation stages. The high AUROC values in the testing and validation datasets reflect the strong discriminatory capability of our model. The consistency of AUROC performance across both datasets underscores the reliability and generalizability of our model. It assures users that the model’s performance is not an artifact of the specific training dataset used but is likely to extend to new, unseen data. This precision is crucial for enriching EHR-derived real-world data that may be used to inform research, improve clinical decision-making, and contribute to improved patient care.

The effectiveness of NLP techniques relies on the availability of high-quality annotated training data. A key strength of this study is the large number of clinically annotated documents used to train and validate the model. Previous studies have used hundreds of annotated examples [11], while our model used several thousand to ensure high model performance when identifying complex medical information.

The model identified 82% of patients with MDD in our clinical population who had a record of anhedonia. The number is similar, if slightly higher, than that reported in other studies which have estimated the prevalence of anhedonia as approximately 75% [34]. The model identified 82% of patients with a record of suicidal thoughts and behavior (including ideation with and without plan or attempt) which is higher than has been reported in a previous study (37.7% for SI, 15.1% for suicidal planning, and 23.7% for suicidal attempts) [2]. The higher prevalence found in the current study may be due to a high proportion of patients in the EHR database receiving secondary or tertiary-level (e.g., inpatient units or specialist services). Indeed, suicidal behaviors were found more commonly among inpatients with MDD compared to patients receiving treatment in other settings [2].

Given the heterogeneous nature of MDD, our model may highlight the differences in frequency of suicidal symptoms between possible subtypes of patients with MDD. Such applications of NLP could be further used to predict other critical clinical outcomes such as adherence or treatment effectiveness. The availability of rich and robust longitudinal, real-world data on specific psychiatric symptoms could then be used to further develop predictive algorithms for determining patients’ risk levels to increase the chances of early interventions before crises occur. A further potential application of our NLP model is the identification of patients for research recruitment; indeed previous studies have used a similar method to identify individuals who were suitable candidates for a study on youth depression [35].

Employing NLP-based approaches to extract symptom data could be questioned when one considers the effectiveness of standardized clinician- or patient-reported measures used in psychiatry to assess the prevalence and severity of symptomology [36]. However, the integration of these standardized assessments into EHRs has been slow due to the associated administrative burden. As such, the use of NLP to extract symptomatic data from unstructured clinical notes is invaluable in assessing symptom burden per visit. This is demonstrated from our analysis of the NeuroBlu Database before and after deploying the model, which found an increase in data availability both from individual patients and the number of visits. This highlights that although standardized measures are becoming more frequently used, clinical notes at present provide a more consistent and frequent source of measuring patient presentation.

The NLP model addresses key challenges in tracking anhedonia and suicidal symptoms by extracting nuanced data from unstructured clinical notes, enriching patient records, and supporting both clinical care and research. By bridging gaps left by structured data, the model provides a continuous view of symptom trajectories, aiding in treatment monitoring and adjustment. Its ability to identify specific symptom clusters allows for more precise cohort selection in studies and trials, enhancing the relevance and impact of research findings. Furthermore, integrating NLP-derived insights with structured metrics offers a comprehensive understanding of patient health, enabling timely and informed interventions, particularly for high-risk symptoms such as suicidal ideation.

Furthermore, our model provides additional granularity of symptoms; structured data was not available to distinguish between suicidal ideation with and without plan/attempt but only the presence of SI. This demonstrates how the application of NLP-based approaches to extract symptom data can improve both the frequency and quality of available data. This study aligns with prior research demonstrating the potential of NLP in mental health care by improving data completeness and clinical insight through unstructured EHR data [4, 29]. Moreover, our approach provides a foundation for broader applications across clinical domains. For instance, in psychiatry, this methodology could be applied to other disorders like anxiety or bipolar disorder to track symptom trajectories and treatment outcomes. Beyond mental health, similar NLP techniques could be employed in domains with substantial unstructured data, such as chronic pain, oncology, or metabolic disorders, to identify clinical trends, predict risk factors, and improve patient care. These applications underline the transformative potential of NLP in advancing personalized and proactive healthcare delivery.

This study is subject to limitations. First, while our model demonstrated strong performance in notes from secondary and tertiary EHRs of patients with MDD, its generalizability to other disease areas, healthcare systems, or patient populations may require further validation and adaptation. Second, the current NLP model has yet to take temporal information of clinical notes into account. This implies that in its current state, should a clinical document describe past suicidal behaviors or anhedonia (i.e., patient presented with suicidal ideation in the past), the model would indicate that suicidal ideation was present within the document. However, except for intake and discharge visits, documentation of clinical presentation is not likely to be focused on distant past behavior, as ongoing documentation is typically focused on current clinical presentation and progress during treatment. It is important to highlight that when identifying anhedonia and suicidal behaviors, clinicians may have a preference around where they document patient symptoms, either structured EHR fields, standardized assessments, or both. Indeed, the ability of the model to enrich the database in the current study was limited by the proportion of patients and visits that had notes available in the EHR record. This highlights the utility of NLP extracted symptom data to compliment, rather than as a substitute for standardized assessments to improve data completeness over time and to create a more holistic picture of the patient journey. Additionally, the reliance on annotated data highlights the resource-intensive nature of developing NLP models, as annotation bottlenecks remain a challenge in the field. Future refinement of the current model may aim to incorporate temporal information and expand validation across diverse datasets and healthcare settings in its output to maximize the clinical utility of the model. Addressing these limitations in future work will enhance the clinical utility of NLP models in psychiatry, enabling even more accurate and timely extraction of critical clinical features. By leveraging these advancements, healthcare providers can move toward truly personalized and proactive mental health care, improving patient outcomes and advancing the integration of data science into routine clinical practice.

## Conclusions

In conclusion, we demonstrated the successful extraction of anhedonia and suicidal symptoms when applying our NLP model to clinical free texts documented in EHRs. While NLP algorithms provide a valuable tool for augmenting clinical insights, our findings also underscore the importance of maintaining structured EHR fields in tandem with NLP techniques. By combining the strengths of both structured and unstructured data analysis, we can provide a more holistic understanding of the MDD disease trajectory with the potential to better equip healthcare providers to deliver timely and effective interventions. The integration of NLP into EHR data analysis offers a pathway toward data-driven, patient-centered mental health care. Advanced NLP models, such as transformers, excel in capturing precise contextual details, enabling earlier detection of risk factors and symptom progression. This precision fosters proactive, tailored interventions, ultimately improving patient outcomes and transforming mental health care delivery.

## Electronic supplementary material

Below is the link to the electronic supplementary material.

Supplementary Material 1

## Data Availability

The data supporting this study originate with Holmusk Technologies, Inc. These de-identified data may be made available upon request and are subject to license agreement with Holmusk. Interested parties should contact publications@holmusk.com to determine licensing terms.

## Abbreviations

AUROC: Area under the receiver operating characteristic
EHR: Electronic health record
ICD-9-CM: International Classification of Diseases, Ninth Revision, Clinical Modification
ICD-10-CM: International Classification of Diseases, Tenth Revision, Clinical Modification
IRR: Interrater reliability
MDD: Major depressive disorder
NLP: Natural language processing
NPP: Negative predictive value
PPV: Positive predictive value
SI: Suicidal ideation

## References

[1] World Health Organization. Preventing suicide: a global imperative [Internet] Geneva 2014 [Available from: http://www.who.int/mental_health/suicide-prevention/world_report_2014/en/

[2] Cai H, Jin Y, Liu S, Zhang Q, Zhang L, Cheung T, et al. Prevalence of suicidal ideation and planning in patients with major depressive disorder: a meta-analysis of observation studies. J Affect Disord. 2021;293:148–58. 34192629 10.1016/j.jad.2021.05.115

[3] Barak-Corren Y, Castro VM, Javitt S, Hoffnagle AG, Dai Y, Perlis RH, et al. Predicting suicidal behavior from Longitudinal Electronic Health Records. Am J Psychiatry. 2017;174(2):154–62. 27609239 10.1176/appi.ajp.2016.16010077

[4] Boggs JM, Kafka JM. A critical review of text mining applications for suicide research. Curr Epidemiol Rep. 2022;9(3):126–34. 35911089 10.1007/s40471-022-00293-w PMC9315081

[5] Fernandes AC, Dutta R, Velupillai S, Sanyal J, Stewart R, Chandran D. Identifying suicide ideation and suicidal attempts in a Psychiatric Clinical Research database using Natural Language Processing. Sci Rep. 2018;8(1):7426. 29743531 10.1038/s41598-018-25773-2 PMC5943451

[6] American Psychiatric Association. The Diagnostic and Statistical Manual of Mental Disorders, Fifth Edition. 5th ed: American Psychiatric Publishing. 2013 May 27, 2013. 991 p.

[7] Khazanov GK, Xu C, Dunn BD, Cohen ZD, DeRubeis RJ, Hollon SD. Distress and anhedonia as predictors of depression treatment outcome: a secondary analysis of a randomized clinical trial. Behav Res Ther. 2020;125:103507. 31896529 10.1016/j.brat.2019.103507 PMC6983353

[8] Whitton AE, Pizzagalli DA. Anhedonia in depression and bipolar disorder. Anhedonia: preclinical, translational, and clinical integration. Springer; 2022. pp. 111–27. 10.1007/7854_2022_323 PMC12665610 35397065

[9] Berk M, Köhler-Forsberg O, Turner M, Penninx B, Wrobel A, Firth J, et al. Comorbidity between major depressive disorder and physical diseases: a comprehensive review of epidemiology, mechanisms and management. World Psychiatry. 2023;22(3):366–87. 37713568 10.1002/wps.21110 PMC10503929

[10] Kshatriya BSA, Nunez NA, Resendez MG, Ryu E, Coombes BJ, Fu S et al. Neural language models with distant supervision to identify major depressive disorder from clinical notes. arXiv Preprint arXiv:210409644. 2021.

[11] Vaci N, Liu Q, Kormilitzin A, De Crescenzo F, Kurtulmus A, Harvey J, et al. Natural language processing for structuring clinical text data on depression using UK-CRIS. BMJ Ment Health. 2020;23(1):21–6. 10.1136/ebmental-2019-300134 PMC10231554 32046989

[12] Borsini A, Wallis ASJ, Zunszain P, Pariante CM, Kempton MJ. Characterizing anhedonia: a systematic review of neuroimaging across the subtypes of reward processing deficits in depression. Cogn Affect Behav Neurosci. 2020;20(4):816–41. 32472419 10.3758/s13415-020-00804-6 PMC7395022

[13] Williams LM. Precision psychiatry: a neural circuit taxonomy for depression and anxiety. Lancet Psychiatry. 2016;3(5):472–80. 27150382 10.1016/S2215-0366(15)00579-9 PMC4922884

[14] Cao B, Zhu J, Zuckerman H, Rosenblat JD, Brietzke E, Pan Z, et al. Pharmacological interventions targeting anhedonia in patients with major depressive disorder: a systematic review. Prog Neuropsychopharmacol Biol Psychiatry. 2019;92:109–17. 30611836 10.1016/j.pnpbp.2019.01.002

[15] Zhu X, Ward J, Cullen B, Lyall DM, Strawbridge RJ, Lyall LM, et al. Phenotypic and genetic associations between anhedonia and brain structure in UK Biobank. Translational Psychiatry. 2021;11(1):395. 34282121 10.1038/s41398-021-01522-4 PMC8289859

[16] Ironside M, Admon R, Maddox SA, Mehta M, Douglas S, Olson DP, et al. Inflammation and depressive phenotypes: evidence from medical records from over 12 000 patients and brain morphology. Psychol Med. 2020;50(16):2790–8. 31615590 10.1017/S0033291719002940 PMC7160032

[17] Walkup JT, Townsend L, Crystal S, Olfson M. A systematic review of validated methods for identifying suicide or suicidal ideation using administrative or claims data. Pharmacoepidemiol Drug Saf. 2012;21(Suppl 1):174–82. 22262604 10.1002/pds.2335

[18] Callahan ST, Fuchs DC, Shelton RC, Balmer LS, Dudley JA, Gideon PS, et al. Identifying suicidal behavior among adolescents using administrative claims data. Pharmacoepidemiol Drug Saf. 2013;22(7):769–75. 23412882 10.1002/pds.3421 PMC3785233

[19] Anderson HD, Pace WD, Brandt E, Nielsen RD, Allen RR, Libby AM, et al. Monitoring suicidal patients in primary care using electronic health records. J Am Board Fam Med. 2015;28(1):65–71. 25567824 10.3122/jabfm.2015.01.140181

[20] Liede A, Wade S, Lethen J, Hernandez RK, Warner D, Abernethy AP, et al. An observational study of concomitant use of emerging therapies and denosumab or zoledronic acid in prostate cancer. Clin Ther. 2018;40(4):536–49. e3. 29395290 10.1016/j.clinthera.2017.12.015

[21] Manemann SM, St Sauver JL, Liu H, Larson NB, Moon S, Takahashi PY, et al. Longitudinal cohorts for harnessing the electronic health record for disease prediction in a US population. BMJ open. 2021;11(6):e044353. 34103314 10.1136/bmjopen-2020-044353 PMC8190051

[22] Patel R, Wee SN, Ramaswamy R, Thadani S, Tandi J, Garg R, et al. NeuroBlu, an electronic health record (EHR) trusted research environment (TRE) to support mental healthcare analytics with real-world data. BMJ Open. 2022;12(4):e057227. 35459671 10.1136/bmjopen-2021-057227 PMC9036423

[23] Crema C, Attardi G, Sartiano D, Redolfi A. Natural language processing in clinical neuroscience and psychiatry: a review. Front Psychiatry. 2022;13:946387. 36186874 10.3389/fpsyt.2022.946387 PMC9515453

[24] Le Glaz A, Haralambous Y, Kim-Dufor DH, Lenca P, Billot R, Ryan TC, et al. Machine Learning and Natural Language Processing in Mental Health: systematic review. J Med Internet Res. 2021;23(5):e15708. 33944788 10.2196/15708 PMC8132982

[25] Malgaroli M, Hull TD, Zech JM, Althoff T. Natural language processing for mental health interventions: a systematic review and research framework. Transl Psychiatry. 2023;13(1):309. 37798296 10.1038/s41398-023-02592-2 PMC10556019

[26] Stade EC, Stirman SW, Ungar LH, Boland CL, Schwartz HA, Yaden DB, et al. Large language models could change the future of behavioral healthcare: a proposal for responsible development and evaluation. Npj Ment Health Res. 2024;3(1):12. 38609507 10.1038/s44184-024-00056-z PMC10987499

[27] Swaminathan A, Lopez I, Mar RAG, Heist T, McClintock T, Caoili K, et al. Natural language processing system for rapid detection and intervention of mental health crisis chat messages. NPJ Digit Med. 2023;6(1):213. 37990134 10.1038/s41746-023-00951-3 PMC10663535

[28] Wu CS, Kuo CJ, Su CH, Wang SH, Dai HJ. Using text mining to extract depressive symptoms and to validate the diagnosis of major depressive disorder from electronic health records. J Affect Disord. 2020;260:617–23. 31541973 10.1016/j.jad.2019.09.044

[29] Spasic I, Nenadic G. Clinical text data in machine learning: systematic review. JMIR Med Inf. 2020;8(3):e17984. 10.2196/17984 PMC7157505 32229465

[30] Kulkarni D, Ghosh A, Girdhari A, Liu S, Vance LA, Unruh M, et al. Enhancing pre-trained contextual embeddings with triplet loss as an effective fine-tuning method for extracting clinical features from electronic health record derived mental health clinical notes. Nat Lang Process J. 2024;6:100045.

[31] Krippendorff K. Reliability in Content Analysis. Hum Commun Res. 2004;30(3):411–33.

[32] Chinchor N, Sundheim BM, editors. MUC-5 evaluation metrics. Fifth Message Understanding Conference (MUC-5): Proceedings of a Conference Held in Baltimore, Maryland, August 25–27, 1993; 1993.

[33] Mandrekar JN. Receiver operating characteristic curve in diagnostic test assessment. J Thorac Oncol. 2010;5(9):1315–6. 20736804 10.1097/JTO.0b013e3181ec173d

[34] Franken IH, Rassin E, Muris P. The assessment of anhedonia in clinical and non-clinical populations: further validation of the Snaith–Hamilton pleasure scale (SHAPS). J Affect Disord. 2007;99(1–3):83–9. 16996138 10.1016/j.jad.2006.08.020

[35] Geraci J, Wilansky P, de Luca V, Roy A, Kennedy JL, Strauss J. Applying deep neural networks to unstructured text notes in electronic medical records for phenotyping youth depression. BMJ Ment Health. 2017;20(3):83–7. 10.1136/eb-2017-102688 PMC5566092 28739578

[36] Kroenke K, Spitzer RL, Williams JB. The PHQ-9: validity of a brief depression severity measure. J Gen Intern Med. 2001;16(9):606–13. 11556941 10.1046/j.1525-1497.2001.016009606.x PMC1495268

