# Supplementary material for: Natural language processing to identify suicidal ideation and anhedonia in major depressive disorder
Source: BMC Med Inform Decis Mak. 2025 Jan 13;25:20. doi: 10.1186/s12911-025-02851-w (PMC11730826; doi:10.1186/s12911-025-02851-w)
Supplement: Supplementary file 1 — Supplementary Material 1 [file 12911_2025_2851_MOESM1_ESM.docx]

**Natural language processing to identify suicidal ideation and anhedonia in major depressive disorder**

Additional File 1

# Additional File 1 – Notes

The 5,000 clinically annotated patient records were checked for duplicates and note length; duplicates and notes shorter than 100 words were removed. The threshold of 100 words was based on clinical judgment as many of these notes appeared to be short notes referring to a previously made longer note, such as “Telemedicine Accompanied [NAME_1] in the clinic during a telemedicine appointment with Dr. [NAME_2] on [DATE_1]. Please see medication management note for details. [NAME_3] response to the service: No SI and HI.”.

List of key words used in NLP development:

SI

Si

si

loss of interest

loss of pleasure

decreased interest.

lost interest

self harm

suicide

suicidal

suicidality

SUICIDE

SUICIDAL

anhedonia

anhydonia

kill himself

kill herself

cut wrist

cut arm

cut herself

cut himself

suicide attempt

choke herself

end her life

end his life

thoughts of dying

overdose

SA

SHI

shi

H/S/I

OD

**Assessment of utility of NLP model for extracting clinical data – Additional details**

Unlike the previous analysis, here we compared the presence of depressive symptoms on a note level rather than on a sentence level. This was to provide a more accurate comparison with structured data. The process of calculating model performance involves parsing notes into sentences, making sentence-level predictions, and subsequently collapsing these predictions to a note-level assessment based on a rule-based aggregation approach. Specifically, we collapse the sentence-level predictions to a note-level prediction using an "any" rule. If any of the sentences within a given note is identified as having a particular symptom, the overall note-level prediction is marked as positive for that symptom. This approach acknowledges that the presence of a symptom in any part of the note is significant for the overall assessment, as it reflects the potential relevance of that symptom to the patient's mental health.

We found no structured data that could distinguish between suicidal ideation with or without intent/plan but only those that captured suicidal ideation more generally therefore for this analysis suicidal ideation with and without intent/plan were collapsed into a single group named suicidality. The structured data pertaining to suicidality we included were clinician observations from the mental status examination (MSE) (Norris et al., 2016) labels specific to suicidal ideation and behavior, any items of the Columbia-Suicide Severity Rating Scale (C-SSRS) (Posner et al., 2011), specific items of the 9-item Patient Health Questionnaire (PHQ-9) (Kroenke et al., 2001), and specific items of the 16-item Quick Inventory of Depressive Symptomology (QIDS-16) ) (Rush et al., 2003). The PHQ-9 and QIDS-16 also include specific items that provide structured data related to anhedonia.

**References**

Kroenke, K., Spitzer, R. L., & Williams, J. B. (2001). The PHQ‐9: validity of a brief depression severity measure. *Journal of general internal medicine*, *16*(9), 606-613.

Norris, D. R., Clark, M. S., & Shipley, S. (2016). The mental status examination. *American family physician*, *94*(8), 635-641.

Posner, K., Brown, G. K., Stanley, B., Brent, D. A., Yershova, K. V., Oquendo, M. A., Currier, G. W., Melvin, G. A., Greenhill, L., & Shen, S. (2011). The Columbia–Suicide Severity Rating Scale: initial validity and internal consistency findings from three multisite studies with adolescents and adults. *American journal of psychiatry*, *168*(12), 1266-1277.

Rush, A. J., Trivedi, M. H., Ibrahim, H. M., Carmody, T. J., Arnow, B., Klein, D. N., Markowitz, J. C., Ninan, P. T., Kornstein, S., & Manber, R. (2003). The 16-Item Quick Inventory of Depressive Symptomatology (QIDS), clinician rating (QIDS-C), and self-report (QIDS-SR): a psychometric evaluation in patients with chronic major depression. *Biological psychiatry*, *54*(5), 573-583.
